# The Auditory Steady-State Response: Electrophysiological Index for Sensory Processing Dysfunction in Psychiatric Disorders

**DOI:** 10.3389/fpsyt.2021.644541

**Published:** 2021-03-11

**Authors:** Shunsuke Sugiyama, Kazutaka Ohi, Ayumi Kuramitsu, Kentaro Takai, Yukimasa Muto, Tomoya Taniguchi, Tomoaki Kinukawa, Nobuyuki Takeuchi, Eishi Motomura, Makoto Nishihara, Toshiki Shioiri, Koji Inui

**Affiliations:** ^1^Department of Psychiatry, Gifu University Graduate School of Medicine, Gifu, Japan; ^2^Department of Anesthesiology, Nagoya University Graduate School of Medicine, Nagoya, Japan; ^3^Depatment of Psychiatry, Aichi Medical University, Nagakute, Japan; ^4^Department of Neuropsychiatry, Mie University Graduate School of Medicine, Tsu, Japan; ^5^Multidisciplinary Pain Center, Aichi Medical University, Nagakute, Japan; ^6^Departmernt of Functioning and Disability, Institute for Developmental Research, Kasugai, Japan

**Keywords:** ASSR, gamma-band oscillation, phase resetting, electroencephalography, magnetoencephalography, schizophrenia, bipolar disorder, autism spectral disorder

## Abstract

Sensory processing is disrupted in several psychiatric disorders, including schizophrenia, bipolar disorder, and autism spectrum disorder. In this review, we focus on the electrophysiological auditory steady-state response (ASSR) driven by high-frequency stimulus trains as an index for disease-associated sensory processing deficits. The ASSR amplitude is suppressed within the gamma band (≥30 Hz) among these patients, suggesting an imbalance between GABAergic and N-methyl-D-aspartate (NMDA) receptor-mediated neurotransmission. The reduced power and synchronization of the 40-Hz ASSR are robust in patients with schizophrenia. In recent years, similar ASSR deficits at gamma frequencies have also been reported in patients with bipolar disorder and autism spectrum disorder. We summarize ASSR abnormalities in each of these psychiatric disorders and suggest that the observed commonalities reflect shared pathophysiological mechanisms. We reviewed studies on phase resetting in which a salient sensory stimulus affects ASSR. Phase resetting induces the reduction of both the amplitude and phase of ASSR. Moreover, phase resetting is also affected by rare auditory stimulus patterns or superimposed stimuli of other modalities. Thus, sensory memory and multisensory integration can be investigated using phase resetting of ASSR. Here, we propose that ASSR amplitude, phase, and resetting responses are sensitive indices for investigating sensory processing dysfunction in psychiatric disorders.

## Introduction

Recent studies have identified multiple shared genetic associations and other commonalities among psychiatric disorders. For example, genome-wide association studies suggest shared molecular pathomechanisms between schizophrenia and bipolar disorder (1, 2), whereas large-scale imaging analyses have revealed similar white matter abnormalities (3) in patients with schizophrenia and bipolar disorders. Recent genetic (2, 4) and neuroimaging studies (5, 6) have also demonstrated shared molecular and neurostructural abnormalities between schizophrenia and autism spectrum disorder. Currently, psychiatric disorders continue to be classified based on observed symptoms rather than underlying pathogenic mechanisms. Classifications such as the International Classification of Diseases (ICD) (7) and the Diagnostic and Statistical Manual of Mental Disorders (DSM) (8) have contributed to the standardization of diagnoses and treatment in clinical practice; however, they provide little information regarding neurobiological mechanisms and treatment targets. Indeed, overemphasis on differential diagnosis according to symptom clusters and clinical history has revealed little about the pathological mechanisms underlying these psychiatric disorders. Therefore, it is important to investigate common biological abnormalities across multiple psychiatric disorders. To address this issue, the National Institute of Mental Health is currently attempting to construct a biological framework for understanding the etiology and symptomology of psychiatric disorders (9).

A common symptom of multiple psychiatric disorders is sensory processing dysfunction (10, 11). Neurophysiological approaches such as magnetoencephalography (MEG) and electroencephalography (EEG) can reveal the electrical activity of neuronal ensembles at high temporal resolution, thereby providing quantitative indices of illness that also reflect disease-associated abnormalities at the cellular level. In this review, we focus on the auditory steady-state response (ASSR), an electrophysiological response driven by a train of stimuli delivered at a sufficiently high rate. ASSR recorded using MEG or EEG has been reported to reach maximum amplitude at approximately 40 Hz (12, 13). Previous MEG (14) and positron-emission tomography (15) studies have reported that ASSR originates in the primary auditory cortex and associated subcortical areas (16). The ASSR has been interpreted as a reflection of oscillatory gamma-band activity representing auditory objects (17–19). Moreover, neural oscillations in the gamma frequency band are believed critical for information processing across cortical networks (20, 21). For example, gamma-band activity increases in the visual (22, 23), auditory (24, 25), and somatosensory cortices (26) in response to modality-specific sensory stimuli. Gamma-band activity is also related to working memory and increases in the hippocampus and prefrontal cortex during memory processing (27–29). Therefore, gamma-band activity is involved in a wide range of brain activities, from low-level sensory processing to higher cognitive functions. Further, ASSR amplitude and phase are believed to reflect the balance between inhibitory GABAergic activity and excitatory glutamatergic activity mediated by the N-methyl-D-aspartate (NMDA) receptor (30–32). Thus, ASSR abnormalities as measured by MEG and EEG can reveal aspects of aberrant neurotransmission and neuronal excitation within specific brain circuits.

In 1999, Kwon et al. first demonstrated that patients with schizophrenia showed reduced power and synchronization of the 40-Hz ASSR (33), and subsequent studies by other groups replicated this finding (34–37). A meta-analysis also concluded that 40-Hz ASSR deficits are robust in schizophrenia (38). These ASSR deficits are consistent with anatomic abnormalities of the auditory cortex observed by magnetic resonance imaging (39, 40). Such ASSR deficits at gamma frequencies have also been discovered in bipolar disorder (41–43) and autism spectrum disorder (44). In this review, we first summarize ASSR abnormalities in each of these psychiatric disorders and discuss the potential commonalities in pathophysiology suggested by these observations. Second, we review studies suggesting that modulation of ASSR amplitude and phase by rare auditory patterns or addition of multimodal stimuli, termed phase resetting, also yield useful index for psychiatric disorders. We propose that ASSR is a sensitive index for investigating sensory memory and multisensory integration deficits in psychiatric disorders.

### ASSR Deficits in Psychiatric Disorders

#### Schizophrenia

Most studies documenting ASSR deficits in schizophrenia have been conducted in the chronic disease phase, suggesting a relationship with symptom expression. Intriguingly, however, reduced evoked power and phase locking of the 40-Hz ASSR have also been documented in first-episode patients (35), high-risk individuals before the onset of psychosis (37), and in first-degree relatives (45, 46). In contrast, individuals with schizotypal personality disorder did not exhibit ASSR deficits (46, 47). These findings suggest that these ASSR deficits reflect pathological development independent of disease course or the side effects of long-term antipsychotic medication.

These ASSR deficits are most consistently observed at 40 Hz, whereas responses are usually intact at 20 and 30 Hz [although reduced ASSR at 30 Hz (35) and enhanced ASSR at 20 Hz (48) have been reported]. Recent studies have also reported impaired evoked ASSR power and phase locking at 80 Hz in schizophrenia (36, 49), and these abnormalities were associated with the severity of hallucinations (36) and negative symptoms, such as flat affect, anhedonia, and poverty of speech (49). Tada et al. reported that deficits in the 40-Hz ASSR during a 300–500 ms train were associated with more severe clinical symptoms and cognitive deficits (37). Moreover, patients with schizophrenia taking new generation antipsychotics exhibited significantly increased 40-Hz ASSR synchronization (45). Collectively, these findings indicate that ASSR may also be a useful quantitative index for current clinical symptoms and treatment response.

#### Bipolar Disorder

Patients with bipolar disorder show a pattern of ASSR deficits similar to that of patients with schizophrenia. To our knowledge, O'Donnell et al. first reported reduced evoked ASSR power at 20, 30, 40, and 50 Hz as well as reduced phase synchronization at 20, 40, and 50 Hz among patients with unmedicated bipolar disorder during manic or mixed episodes using EEG (41). Such ASSR deficits have also been documented in depressive (42), euthymic (43), and manic (41) states in the first episode (35) and the chronic state (41–43) and in both medicated (42, 43) and unmedicated patients (41).

In contrast to a comparative group of patients with bipolar disorder, no ASSR deficits were observed in a parallel group with major depressive disorder (50). In fact, to our knowledge, only one study has reported ASSR deficits in major depressive disorder (51), and reduced ASSR power was found at 30 Hz but not at 40 Hz as observed in patients with bipolar disorder and schizophrenia (51). These findings suggest that major depressive disorder and bipolar disorder have distinct neurophysiological bases and further that 40-Hz ASSR can be used to distinguish bipolar disorder from major depressive disorder (50).

#### Autism Spectrum Disorder

Wilson et al. first reported reduced 40-Hz ASSR power in 7–17-year-old children and adolescents with autism using MEG (52), with a greater reduction in the left hemisphere. Thereafter, Rojas et al. found reduced evoked power and phase locking of left and right 40-Hz ASSRs among both adults with autism and parents of children with autism (53), suggesting that ASSR is a useful index for diagnosis and risk evaluation. However, utility may be limited to adults as ASSR amplitude increases from childhood through adolescence and plateaus in early adulthood (54). Further, no significant deficits in 20- and 40-Hz ASSRs were found among 5–7-year-old children with autism spectrum disorder (55).

#### Shared Pathophysiology Among Psychiatric Disorders

Patients with schizophrenia, bipolar disorder, and autism spectrum disorder demonstrate similar patterns of ASSR deficits, suggesting shared neural circuit dysfunction. One emerging hypothesis is that ASSR deficits reflect dysfunction of the GABAergic and/or NMDAergic systems. Blockers of NMDA receptors, such as phencyclidine and ketamine, evoke psychotic symptoms in healthy individuals, exacerbate positive symptoms in patients with schizophrenia, and induce various schizotypic electrophysiological and behavioral abnormalities in experimental animal models (56). For instance, Sohal et al. demonstrated that optogenetic downregulation of parvalbumin-positive GABAergic interneuron activity in mice reduced gamma-band oscillations (57), whereas Sivarao et al. reported that the 40-Hz ASSR in awake rats depended on the degree of NMDA receptor channel blockade (30). Collectively, these findings are consistent with evidence implicating GABA (58) and/or NMDA (59) transmission impairment in schizophrenia.

Post-mortem brain studies of patients with schizophrenia and bipolar disorder have also reported reduced interneuron density in the cerebral cortex and hippocampus (60). Similar to the GABAergic dysfunction in bipolar disorder is the therapeutic efficacy of the mood stabilizer valproate, which has been shown to increase GABA turnover in rat brain (61). Moreover, valproate has been reported to increase GABA levels in human plasma, suggesting that it enhances GABA activity in the central nervous system (62). However, poor understanding of the mechanism of action of valproate in bipolar disorder is a limitation (63), and valproate is not effective in treating schizophrenia or autism spectrum disorder, despite sharing the GABAergic dysfunction hypothesis. A recent study of induced pluripotent stem cell-derived organoids from patients with schizophrenia and bipolar disorder found enhanced GABAergic specification (64), suggesting that the reduction in GABAergic neurons observed after disease onset is a compensatory response to maintain the excitatory/inhibitory balance within neural circuits during cortical development.

Conversely, 40-Hz ASSR deficits have not been observed in patients with major depressive disorder. Hirano et al. showed that spontaneous gamma band activity is high in patients with schizophrenia and that the degree of 40-Hz ASSR deficits was associated with increased spontaneous gamma-band activity (65). Moreover, ketamine, an NMDA receptor antagonist, was effective in treating depression (66) and increases resting-state gamma-band activity (67). Therefore, patients with major depressive disorder, in contrast to those with schizophrenia, may have reduced spontaneous gamma-band activity, and consequently, ASSR deficits may not have been observed. However, spontaneous gamma-band activity has not yet been investigated in patients with major depressive disorder. The number of reports on ASSR in major depressive disorder is small, and similarities and differences with other diseases that have ASSR deficits need to be discussed in the future.

Dysfunction of the GABAergic system has also been implicated in autism spectrum disorder. For example, multiple mouse models of autism established via toxins or manipulation of associated genes exhibit reduced number of neocortical parvalbumin-positive inhibitory neurons (68). A post-mortem study also reported reduced GABA-synthesizing enzymes in parietal and cerebellar cortices of patients with autism spectrum disorder (69), whereas a proton magnetic resonance spectroscopy study reported reduced GABA concentration in the auditory and frontal cortices of living patients (70). These GABAergic deficits may result in a relative excess of glutamatergic activity. Indeed, Fatemi's hyper-glutamatergic hypothesis of autism spectrum disorder posits that deficits in GABA-synthesizing enzymes and increased GABA uptake by astrocytes led to excess cortical glutamate (71).

Autism spectrum disorder and schizophrenia also share behavioral symptoms such as difficulties with social cognition, social interaction, and executive functions (72). In fact, autism spectrum disorder was initially believed to be an early stage of schizophrenia (73). Furthermore, an altered ratio of excitatory to inhibitory cortical activity has been reported in both autism spectrum disorder and schizophrenia (74). Yizhar et al. demonstrated that psychosocial dysfunction, a trait common to both disorders, was associated with increased excitation/inhibition ratio in mouse prefrontal cortex (75). Therefore, understanding the causes of excitation/inhibition imbalance could provide clues to the pathophysiology of these disorders as well as to novel treatment strategies. Further, ASSR could be a sensitive electrophysiological indicator reflecting the excitation/inhibition imbalance common among schizophrenia, bipolar disorder, and autism spectrum disorder.

### Perspectives on Neurophysiological Research Using Phase Resetting of ASSR

Phase resetting is a phenomenon that occurs when a stimulus perturbs the phase within a neural oscillation. Resetting the phase of ongoing neural oscillation induces the synchronization of different neurons or brain regions (76). Phase resetting is the fundamental mechanism underlying synchronization, and neural synchronization is believed to play a role in information processing (77), neuronal communication (78), motor coordination (79), and memory (80). For example, in clinical research, epilepsy is considered a disease that results from neuronal hyper-synchronization (81). The generation of resting tremor in Parkinson's disease has been suggested to be owing to abnormal synchronization of neuronal activity (82). In schizophrenia, the disruption of neural synchronization is believed to be related to fragmented cognitive experience (83).

**Figure 1:**
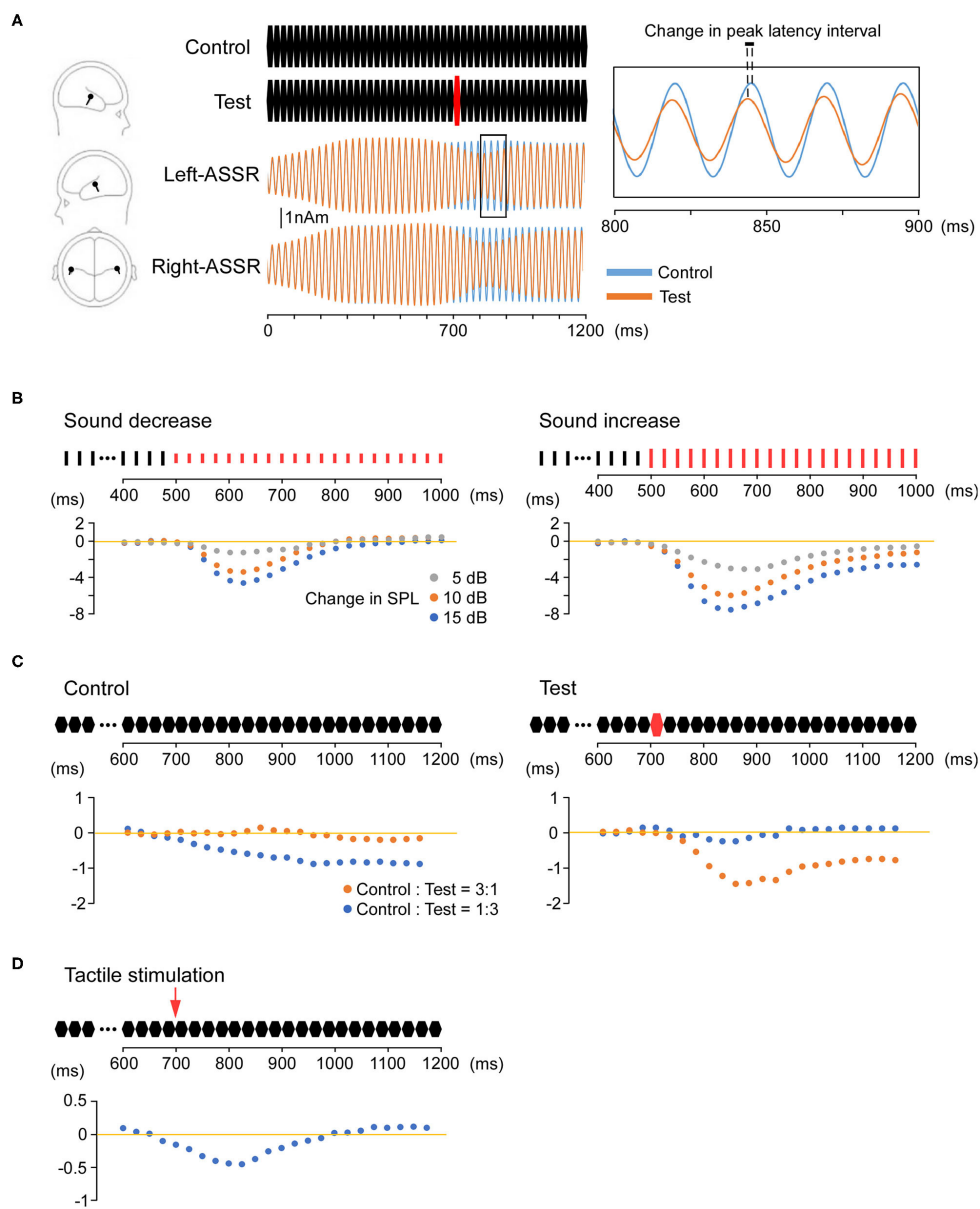
Phase resetting of the auditory steady-state response (ASSR). **(A)** Modulation of the ASSR by stimulus intensity (sound pressure). Repeated presentation of a 25-ms pure tone (upper middle) elicits the 40-Hz ASSR. An abrupt increase in sound pressure at 700 ms causes a reduction in amplitude and phase (phase resetting). The location of estimated dipoles (left panel), source-strength waveforms (middle), and enlarged waveforms on an expanded time axis (right) are also shown. **(B)** Increasing sound pressure reduces ASSR latency. The Y-axis shows changes in the peak latency interval over time relative to the control condition. The control stimulus is a 1,000-ms train of clicks at 40 Hz. The test stimuli are a 500-ms train of clicks identical to the control stimulus and a subsequent 500-ms click-train of the same frequency but altered sound pressure compared with the control stimulus (−5, −10, −15, 5, 10, or 15 dB). The degree of phase resetting depends on the magnitude of the sound pressure change. **(C)** Modulation of the ASSR by deviant stimuli (odd ball condition). The Y-axis shows changes in the peak latency interval over time compared with the control-only (left) and test-only (right) conditions. The control stimulus is a 1,200-ms train of 25-ms pure tones. The test stimulus is a similar train of pure tones in which the tone sound pressure at 700 ms is increased by 15 dB. Under an oddball paradigm, phase resetting is observed when either the control or test stimulus is rare (deviant). **(D)** Modulation of the ASSR by multimodal stimulation. The Y-axis shows changes in the peak latency interval over time compared with the control condition. As the test stimulus, an electrical pulse is presented to the dorsum of the left or right hand at 700 ms during the train of 25-ms pure tones. Tactile stimulation causes phase resetting of the ASSR, and this cross-modal effect is observed from approximately 50–125 ms after the onset of tactile stimulation.

A salient sensory stimulus on ASSR causes phase resetting that modulates the amplitude and phase (Figure 1A) Rohrbaugh et al. first reported that a foreground auditory stimulus reduced both the amplitude and latency of a 40-Hz ASSR evoked by a background rhythmic probe stimulus (84–86). In addition, phase resetting of the 40-Hz ASSR has been reported following a sudden change in stimulus frequency or intensity (87). In a study using an oddball paradigm, button pressing in response to a rare stimulus also caused phase resetting of the 40-Hz ASSR (88). Furthermore, Ross et al. reported that the ASSR was modulated by changing stimulus onset (19), violating the periodicity of a sound stimulus (89), and introducing an interfering stimulus (90). These findings suggest that perturbing stimuli reset the oscillations and shift the ASSR phase back to that of the driving source (90).

Our recent study indicated that increasing the sound pressure can induce a proportionate reduction in ASSR latency (Figure 1B) (91). We also demonstrated that ASSR latency can be shortened without changing the physical characteristics of the peripheral input (92). Using an oddball paradigm, we found that a control stimulus with unchanging sequence shortened the ASSR latency when presented with a low probability among other stimulus patterns (Figure 1C). These findings indicate that ASSR phase resetting can be induced by an intrinsic comparison process based on sensory memory. Sensory memory impairment has been reported in several neurological and psychiatric disorders, primarily using mismatch negativity (MMN) (93), a negative component of the event-related potential elicited by a deviant stimulus embedded in repetitive stimuli (an oddball paradigm), with maximum negativity at Fz and positivity at the mastoid (94). Mismatch negativity reflects the automatic change detection process based on short-term sensory memory and thus serves as an index of sensory memory disruption (95). For example, patients with schizophrenia (96, 97), autism spectrum disorder (98), and Alzheimer's disease (99) have all demonstrated smaller auditory MMN waveforms than healthy controls. Although previous studies have reported that ASSR is modulated by selective attention (100, 101), our paradigms (91, 92), such as oddball paradigms which are typically used to detect MMN, do not require conditions of attention. Changes in ASSR during such odd ball paradigms (91, 92) may facilitate efficient assessment of sensory memory impairments in psychiatric disorders because such measurements do not require multiple stimulus repetitions, thereby reducing experimental time and patient burden.

We also recently demonstrated reduced ASSR latency by simultaneous tactile stimulation (Figure 1D) (102), strongly suggesting that cross-modal input increases the speed of ongoing auditory processing. This cross-modal ASSR paradigm may thus permit the assessment of multimodal sensory integration with high test–retest reliability (103). Moreover, the 40-Hz ASSR is considered superior for providing information on processing speed compared with other sensory paradigms because peak latency can be measured reliably every 12.5 ms. Indeed, our findings of reduced ASSR latency during multimodal stimulation are consistent with previous studies demonstrating faster object recognition using both auditory and visual features compared with either modality alone and with the appearance of unique early-onset multimodal ERP waveforms originating from both sensory and frontal cortex (104, 105). Although previous studies have shown impaired multisensory integration in patients with schizophrenia (106) and autism spectrum disorder (107), psychophysical rather than neurophysiological indicators were assessed. We suggest that the ASSR serves as a robust electrophysiological index of multisensory integration deficits in psychiatric disorders.

## Conclusion

Patients with schizophrenia, bipolar disorder, and autism spectrum disorder all exhibit deficits in the ASSR at gamma-band frequencies, suggesting shared pathomechanisms including dysregulation of cortical excitatory/inhibitory balance. Moreover, ASSR magnitude and phase reflect auditory memory, multimodal sensory integration, and the comparison of incoming sensory stimuli with previous memory traces. Thus, ASSR could be a sensitive electrophysiological index for sensory processing deficits in psychiatric disorders.

## Author Contributions

SS conducted the literature review, SS and KI drafted the manuscript, SS and EM created the figure, AK, KT, YM, TT, TK, NT, MN, and TS provided valuable critical input on the manuscript, and KO edited the final draft. All authors contributed to the article and approved the submitted version.

## Conflict of Interest

The authors declare that the research was conducted in the absence of any commercial or financial relationships that could be construed as a potential conflict of interest.

## References

[1] Lee SH Ripke SH Neale BM Faraone SV Purcell SM Perlis RH. Genetic relationship between five psychiatric disorders estimated from genome-wide SNPs. Nat Genet. (2013) 45:984–94. 10.1038/ng.2711 23933821 PMC3800159

[2] Cross-Disorder Group of the Psychiatric Genomics Consortium. Identification of risk loci with shared effects on five major psychiatric disorders: a genome-wide analysis. Lancet. (2013) 381:1371–9. 10.1016/S0140-6736(12)62129-1 23453885 PMC3714010

[3] Koshiyama D Fukunaga M Okada N Morita K Nemoto K Usui K. White matter microstructural alterations across four major psychiatric disorders: mega-analysis study in 2937 individuals. Mol Psychiatry. (2020) 25:883–95. 10.1038/s41380-019-0553-7 31780770 PMC7156346

[4] Geschwind DH Flint J. Genetics and genomics of psychiatric disease. Science. (2015) 349:1489–94. 10.1126/science.aaa8954 26404826 PMC4694563

[5] Pinkham AE Hopfinger JB Pelphrey KA Piven J Penn DL. Neural bases for impaired social cognition in schizophrenia and autism spectrum disorders. Schizophr Res. (2008) 99:164–75. 10.1016/j.schres.2007.10.024 18053686 PMC2740744

[6] Sugranyes G Kyriakopoulos M Corrigall R Taylor E Frangou S. Autism spectrum disorders and schizophrenia: meta-analysis of the neural correlates of social cognition. PLOS ONE. (2011) 6:e25322. 10.1371/journal.pone.0025322 21998649 PMC3187762

[7] World Health Organization. International Classification of Diseases ICD-10. 10th ed. Geneva: World Health Organization (1992).

[8] American Psychiatric Association. Diagnostic and Statistical Manual of Mental Disorders DSM-5. Washington, DC: American Psychiatric Association (2013).

[9] Insel T Cuthbert B Garvey M Heinssen R Pine DS Quinn K. Research domain criteria (RDoC): toward a new classification framework for research on mental disorders. Am J Psychiatry. (2010) 167:748–51. 10.1176/appi.ajp.2010.09091379 20595427

[10] Kas MJ Fernandes C Schalkwyk LC Collier DA. Genetics of behavioural domains across the neuropsychiatric spectrum; of mice and men. Mol Psychiatry. (2007) 12:324–30. 10.1038/sj.mp.4001979 17389901

[11] Harrison LA Kats A Williams ME Aziz-Zadeh L. The importance of sensory processing in mental health: a proposed addition to the research domain criteria (RDoC) and suggestions for RDoC 2.0. Front Psychol. (2019) 10:103. 10.3389/fpsyg.2019.00103 30804830 PMC6370662

[12] Galambos R Makeig S Talmachoff PJ. A 40-Hz auditory potential recorded from the human scalp. Proc Natl Acad Sci USA. (1981) 78:2643–7. 10.1073/pnas.78.4.2643 6941317 PMC319406

[13] Ross B Borgmann C Draganova R Roberts LE Pantev C. A high-precision magnetoencephalographic study of human auditory steady-state responses to amplitude-modulated tones. J Acoust Soc Am. (2000) 108:679–91. 10.1121/1.429600 10955634

[14] Ross B. A novel type of auditory responses: temporal dynamics of 40-Hz steady-state responses induced by changes in sound localization. J Neurophysiol. (2008) 100:1265–77. 10.1152/jn.00048.2008 18632891

[15] Pastor MA Artieda J Arbizu J Marti-Climent JM Peñuelas I Masdeu JC. Activation of human cerebral and cerebellar cortex by auditory stimulation at 40 Hz. J Neurosci. (2002) 22:10501–6. 10.1523/JNEUROSCI.22-23-10501.2002 12451150 PMC6758739

[16] Herdman AT Lins O Van Roon P Stapells DR Scherg M Picton TW. Intracerebral sources of human auditory steady-state responses. Brain Topogr. (2002) 15:69–86. 10.1023/A:1021470822922 12537303

[17] Santarelli R Maurizi M Conti G Ottaviani F Paludetti G Pettorossi VE. Generation of human auditory steady-state responses (SSRs). II: addition of responses to individual stimuli. Hear Res. (1995) 83:9–18. 10.1016/0378-5955(94)00185-S 7607994

[18] Santarelli R Conti G. Generation of auditory steady-state responses: linearity assessment. Scand Audiol Suppl. (1999) 51:23–32. 10803911

[19] Ross B Picton TW Pantev C. Temporal integration in the human auditory cortex as represented by the development of the steady-state magnetic field. Hear Res. (2002) 165:68–84. 10.1016/S0378-5955(02)00285-X 12031517

[20] Uhlhaas PJ Haenschel C Nikolić D Singer W. The role of oscillations and synchrony in cortical networks and their putative relevance for the pathophysiology of schizophrenia. Schizophr Bull. (2008) 34:927–43. 10.1093/schbul/sbn062 18562344 PMC2632472

[21] Bosman CA Lansink CS Pennartz CM. Functions of gamma-band synchronization in cognition: from single circuits to functional diversity across cortical and subcortical systems. Eur J Neurosci. (2014) 39:1982–99. 10.1111/ejn.12606 24809619

[22] Gray CM König P Engel AK Singer W. Oscillatory responses in cat visual cortex exhibit inter-columnar synchronization which reflects global stimulus properties. Nature. (1989) 338:334–7. 10.1038/338334a0 2922061

[23] Tallon-Baudry C Bertrand O. Oscillatory gamma activity in humans and its role in object representation. Trends Cogn Sci. (1999) 3:151–62. 10.1016/S1364-6613(99)01299-1 10322469

[24] Fukushima M Saunders RC Leopold DA Mishkin M Averbeck BB. Spontaneous high-gamma band activity reflects functional organization of auditory cortex in the awake macaque. Neuron. (2012) 74:899–910. 10.1016/j.neuron.2012.04.014 22681693 PMC3372858

[25] Polomac N Leicht G Nolte G Andreou C Schneider TR Steinmann S. Generators and connectivity of the early auditory evoked gamma band response. Brain Topogr. (2015) 28:865–78. 10.1007/s10548-015-0434-6 25926268

[26] Faivre N Dönz J Scandola M Dhanis H Bello Ruiz J Bernasconi F. Self-grounded vision: hand ownership modulates visual location through cortical β and γ oscillations. J Neurosci. (2017) 37:11–22. 10.1523/JNEUROSCI.0563-16.2016 28053026 PMC6705670

[27] Carr MF Karlsson MP Frank LM. Transient slow gamma synchrony underlies hippocampal memory replay. Neuron. (2012) 75:700–13. 10.1016/j.neuron.2012.06.014 22920260 PMC3428599

[28] Lundqvist M Rose J Herman P Brincat SL Buschman TJ Miller EK. Gamma and beta bursts underlie working memory. Neuron. (2016) 90:152–64. 10.1016/j.neuron.2016.02.028 26996084 PMC5220584

[29] Yamamoto J Suh J Takeuchi D Tonegawa S. Successful execution of working memory linked to synchronized high-frequency gamma oscillations. Cell. (2014) 157:845–57. 10.1016/j.cell.2014.04.009 24768692

[30] Sivarao DV Chen P Senapati A Yang Y Fernandes A Benitex Y. 40 Hz Auditory steady-state response is a pharmacodynamic biomarker for cortical NMDA receptors. Neuropsychopharmacology. (2016) 41:2232–40. 10.1038/npp.2016.17 26837462 PMC4946051

[31] Light GA Zhang W Joshi YB Bhakta S Talledo JA Swerdlow NR. Single-dose memantine improves cortical oscillatory response dynamics in patients with schizophrenia. Neuropsychopharmacology. (2017) 42:2633–9. 10.1038/npp.2017.81 28425497 PMC5686499

[32] Tada M Kirihara K Koshiyama D Fujioka M Usui K Uka T. Gamma-band auditory steady-state response as a neurophysiological marker for excitation and inhibition balance: a review for understanding schizophrenia and other neuropsychiatric disorders. Clin EEG Neurosci. (2020) 51:234–43. 10.1177/1550059419868872 31402699

[33] Kwon JS O'Donnell BF Wallenstein GV Greene RW Hirayasu Y Nestor PG. Gamma frequency range abnormalities to auditory stimulation in schizophrenia. Arch Gen Psychiatry. (1999) 56:1001–5. 10.1001/archpsyc.56.11.1001 10565499 PMC2863027

[34] Light GA Hsu JL Hsieh MH Meyer-Gomes K Sprock J Swerdlow NR. Gamma band oscillations reveal neural network cortical coherence dysfunction in schizophrenia patients. Biol Psychiatry. (2006) 60:1231–40. 10.1016/j.biopsych.2006.03.055 16893524

[35] Spencer KM Salisbury DF Shenton ME McCarley RW. Gamma-band auditory steady-state responses are impaired in first episode psychosis. Biol Psychiatry. (2008) 64:369–75. 10.1016/j.biopsych.2008.02.021 18400208 PMC2579257

[36] Tsuchimoto R Kanba S Hirano S Oribe N Ueno T Hirano Y. Reduced high and low frequency gamma synchronization in patients with chronic schizophrenia. Schizophr Res. (2011) 133:99–105. 10.1016/j.schres.2011.07.020 21849245

[37] Tada M Nagai T Kirihara K Koike S Suga M Araki T. Differential alterations of auditory gamma oscillatory responses between pre-onset high-risk individuals and first-episode schizophrenia. Cereb Cortex. (2016) 26:1027–35. 10.1093/cercor/bhu278 25452567

[38] Thuné H Recasens M Uhlhaas PJ. The 40-Hz auditory steady-state response in patients with schizophrenia: a meta-analysis. JAMA Psychiatry. (2016) 73:1145–53. 10.1001/jamapsychiatry.2016.2619 27732692

[39] Wright IC Rabe-Hesketh S Woodruff PW David AS Murray RM Bullmore ET. Meta-analysis of regional brain volumes in schizophrenia. Am J Psychiatry. (2000) 157:16–25. 10.1176/ajp.157.1.16 10618008

[40] Honea R Crow TJ Passingham D Mackay CE. Regional deficits in brain volume in schizophrenia: a meta-analysis of voxel-based morphometry studies. Am J Psychiatry. (2005) 162:2233–45. 10.1176/appi.ajp.162.12.2233 16330585

[41] O'Donnell BF Hetrick WP Vohs JL Krishnan GP Carroll CA Shekhar A. Neural synchronization deficits to auditory stimulation in bipolar disorder. NeuroReport. (2004) 15:1369–72. 10.1097/01.wnr.0000127348.64681.b2 15167568

[42] Oda Y Onitsuka T Tsuchimoto R Hirano S Oribe N Ueno T. Gamma band neural synchronization deficits for auditory steady state responses in bipolar disorder patients. PLOS ONE. (2012) 7:e39955. 10.1371/journal.pone.0039955 22792199 PMC3390322

[43] Rass O Krishnan G Brenner CA Hetrick WP Merrill CC Shekhar A. Auditory steady state response in bipolar disorder: relation to clinical state, cognitive performance, medication status, and substance disorders. Bipolar Disord. (2010) 12:793–803. 10.1111/j.1399-5618.2010.00871.x 21176026 PMC3060563

[44] Rojas DC Wilson LB. γ-band abnormalities as markers of autism spectrum disorders. Biomark Med. (2014) 8:353–68. 10.2217/bmm.14.15 24712425 PMC4105225

[45] Hong LE Summerfelt A McMahon R Adami H Francis G Elliott A. Evoked gamma band synchronization and the liability for schizophrenia. Schizophr Res. (2004) 70:293–302. 10.1016/j.schres.2003.12.011 15329305

[46] Rass O Forsyth JK Krishnan GP Hetrick WP Klaunig MJ Breier A. Auditory steady state response in the schizophrenia, first-degree relatives, and schizotypal personality disorder. Schizophr Res. (2012) 136:143–9. 10.1016/j.schres.2012.01.003 22285558 PMC3298621

[47] Brenner CA Sporns O Lysaker PH O'Donnell BF. EEG synchronization to modulated auditory tones in schizophrenia, schizoaffective disorder, and schizotypal personality disorder. Am J Psychiatry. (2003) 160:2238–40. 10.1176/appi.ajp.160.12.2238 14638599

[48] Vierling-Claassen D Siekmeier P Stufflebeam S Kopell N. Modeling GABA alterations in schizophrenia: a link between impaired inhibition and altered gamma and beta range auditory entrainment. J Neurophysiol. (2008) 99:2656–71. 10.1152/jn.00870.2007 18287555 PMC2679675

[49] Hamm JP Gilmore CS Picchetti NA Sponheim SR Clementz BA. Abnormalities of neuronal oscillations and temporal integration to low- and high-frequency auditory stimulation in schizophrenia. Biol Psychiatry. (2011) 69:989–96. 10.1016/j.biopsych.2010.11.021 21216392 PMC3174270

[50] Isomura S Onitsuka T Tsuchimoto R Nakamura I Hirano S Oda Y. Differentiation between major depressive disorder and bipolar disorder by auditory steady-state responses. J Affect Disord. (2016) 190:800–6. 10.1016/j.jad.2015.11.034 26625092

[51] Chen J Gong Q Wu F. Deficits in the 30-Hz auditory steady-state response in patients with major depressive disorder. NeuroReport. (2016) 27:1147–52. 10.1097/WNR.0000000000000671 27563737

[52] Wilson TW Rojas DC Reite ML Teale PD Rogers SJ. Children and adolescents with autism exhibit reduced MEG steady-state gamma responses. Biol Psychiatry. (2007) 62:192–7. 10.1016/j.biopsych.2006.07.002 16950225 PMC2692734

[53] Rojas DC Maharajh K Teale P Rogers SJ. Reduced neural synchronization of gamma-band MEG oscillations in first-degree relatives of children with autism. BMC Psychiatry. (2008) 8:66. 10.1186/1471-244X-8-66 18673566 PMC2518921

[54] Rojas DC Maharajh K Teale PD Kleman MR Benkers TL Carlson JP. Development of the 40Hz steady state auditory evoked magnetic field from ages 5 to 52. Clin Neurophysiol. (2006) 117:110–7. 10.1016/j.clinph.2005.08.032 16316780

[55] Ono Y Kudoh K Ikeda T Takahashi T Yoshimura Y Minabe Y. Auditory steady-state response at 20 Hz and 40 Hz in young typically developing children and children with autism spectrum disorder. Psychiatry Clin Neurosci. (2020) 74:354–61. 10.1111/pcn.12998 32155301

[56] Jentsch JD Roth RH. The neuropsychopharmacology of phencyclidine: from NMDA receptor hypofunction to the dopamine hypothesis of schizophrenia. Neuropsychopharmacology. (1999) 20:201–25. 10.1016/S0893-133X(98)00060-8 10063482

[57] Sohal VS Zhang F Yizhar O Deisseroth K. Parvalbumin neurons and gamma rhythms enhance cortical circuit performance. Nature. (2009) 459:698–702. 10.1038/nature07991 19396159 PMC3969859

[58] Lewis DA Curley AA Glausier JR Volk DW. Cortical parvalbumin interneurons and cognitive dysfunction in schizophrenia. Trends Neurosci. (2012) 35:57–67. 10.1016/j.tins.2011.10.004 22154068 PMC3253230

[59] Kantrowitz JT Javitt DC. N-methyl-D-aspartate (NMDA) receptor dysfunction or dysregulation: the final common pathway on the road to schizophrenia? Brain Res Bull. (2010) 83:108–21. 10.1016/j.brainresbull.2010.04.006 20417696 PMC2941541

[60] Benes FM Berretta S. GABAergic interneurons: implications for understanding schizophrenia and bipolar disorder. Neuropsychopharmacology. (2001) 25:1–27. 10.1016/S0893-133X(01)00225-1 11377916

[61] Löscher W. Valproate enhances GABA turnover in the substantia nigra. Brain Res. (1989) 501:198–203. 10.1016/0006-8993(89)91044-5 2508993

[62] Shiah IS Yatham LN Baker GB. Divalproex sodium increases plasma GABA levels in healthy volunteers. Int Clin Psychopharmacol. (2000) 15:221–5. 10.1097/00004850-200015040-00005 10954062

[63] Rosenberg G. The mechanisms of action of valproate in neuropsychiatric disorders: can we see the forest for the trees? Cell Mol Life Sci. (2007) 64:2090–103. 10.1007/s00018-007-7079-x 17514356 PMC11149473

[64] Sawada T Chater TE Sasagawa Y Yoshimura M Fujimori-Tonou N Tanaka K. Developmental excitation-inhibition imbalance underlying psychoses revealed by single-cell analyses of discordant twins-derived cerebral organoids. Mol Psychiatry. (2020) 25:2695–711. 10.1038/s41380-020-0844-z 32764691 PMC7577852

[65] Hirano Y Oribe N Kanba S Onitsuka T Nestor PG Spencer KM. Spontaneous gamma activity in schizophrenia. JAMA Psychiatry. (2015) 72:813–21. 10.1001/jamapsychiatry.2014.2642 25587799 PMC4768724

[66] Zarate CA Jr Singh JB Carlson PJ Brutsche NE Ameli R Luckenbaugh DA. A randomized trial of an N-methyl-D-aspartate antagonist in treatment-resistant major depression. Arch Gen Psychiatry. (2006) 63:856–64. 10.1001/archpsyc.63.8.856 16894061

[67] Rivolta D Heidegger T Scheller B Sauer A Schaum M Birkner K. Ketamine dysregulates the amplitude and connectivity of high-frequency oscillations in cortical-subcortical networks in humans: evidence from resting-state magnetoencephalography-recordings. Schizophr Bull. (2015) 41:1105–14. 10.1093/schbul/sbv051 25987642 PMC4535642

[68] Gogolla N Leblanc JJ Quast KB Südhof TC Fagiolini M Hensch TK. Common circuit defect of excitatory-inhibitory balance in mouse models of autism. J Neurodev Disord. (2009) 1:172–81. 10.1007/s11689-009-9023-x 20664807 PMC2906812

[69] Fatemi SH Halt AR Stary JM Kanodia R Schulz SC Realmuto GR. Glutamic acid decarboxylase 65 and 67 kDa proteins are reduced in autistic parietal and cerebellar cortices. Biol Psychiatry. (2002) 52:805–10. 10.1016/S0006-3223(02)01430-0 12372652

[70] Rojas DC Singel D Steinmetz S Hepburn S Brown MS. Decreased left perisylvian GABA concentration in children with autism and unaffected siblings. Neuroimage. (2014) 86:28–34. 10.1016/j.neuroimage.2013.01.045 23370056 PMC3773530

[71] Fatemi SH. The hyperglutamatergic hypothesis of autism. Prog Neuropsychopharmacol Biol Psychiatry. (2008) 32:911, author reply 912–3. 10.1016/j.pnpbp.2007.11.005 18160196

[72] Cheung C Yu K Fung G Leung M Wong C Li Q. Autistic disorders and schizophrenia: related or remote? An anatomical likelihood estimation. PLOS ONE. (2010) 5:e12233. 10.1371/journal.pone.0012233 20805880 PMC2923607

[73] Kolvin I. Studies in the childhood psychoses. I. Diagnostic criteria and classification. Br J Psychiatry. (1971) 118:381–4. 10.1192/bjp.118.545.381 5576635

[74] Gao R Penzes P. Common mechanisms of excitatory and inhibitory imbalance in schizophrenia and autism spectrum disorders. Curr Mol Med. (2015) 15:146–67. 10.2174/1566524015666150303003028 25732149 PMC4721588

[75] Yizhar O Fenno LE Prigge M Schneider F Davidson TJ O'Shea DJ. Neocortical excitation/inhibition balance in information processing and social dysfunction. Nature. (2011) 477:171–8. 10.1038/nature10360 21796121 PMC4155501

[76] Varela F Lachaux JP Rodriguez E Martinerie J. The brainweb: phase synchronization and large-scale integration. Nat Rev Neurosci. (2001) 2:229–39. 10.1038/35067550 11283746

[77] Singer W. Synchronization of cortical activity and its putative role in information processing and learning. Annu Rev Physiol. (1993) 55:349–74. 10.1146/annurev.ph.55.030193.002025 8466179

[78] Fries P. A mechanism for cognitive dynamics: neuronal communication through neuronal coherence. Trends Cogn Sci. (2005) 9:474–80. 10.1016/j.tics.2005.08.011 16150631

[79] Schnitzler A Gross J. Normal and pathological oscillatory communication in the brain. Nat Rev Neurosci. (2005) 6:285–96. 10.1038/nrn1650 15803160

[80] Fell J Axmacher N. The role of phase synchronization in memory processes. Nat Rev Neurosci. (2011) 12:105–18. 10.1038/nrn2979 21248789

[81] Netoff TI Clewley R Arno S Keck T White JA. Epilepsy in small-world networks. J Neurosci. (2004) 24:8075–83. 10.1523/JNEUROSCI.1509-04.2004 15371508 PMC6729784

[82] Hurtado JM Lachaux JP Beckley DJ Gray CM Sigvardt KA. Inter- and intralimb oscillator coupling in parkinsonian tremor. Mov Disord. (2000) 15:683–91. 10.1002/1531-8257(200007)15:4<683::aid-mds1013>3.0.co;2-# 10928579

[83] Tononi G Edelman GM. Schizophrenia and the mechanisms of conscious integration. Brain Res Brain Res Rev. (2000) 31:391–400. 10.1016/S0165-0173(99)00056-9 10719167

[84] Rohrbaugh JW Varner JL Paige SR Eckardt MJ Ellingson RJ. Event-related perturbations in an electrophysiological measure of auditory function: a measure of sensitivity during orienting? Biol Psychol. (1989) 29:247–71. 10.1016/0301-0511(89)90022-7 2640160

[85] Rohrbaugh JW Varner JL Paige SR Eckardt MJ Ellingson RJ. Auditory and visual event-related perturbations in the 40 Hz auditory steady-state response. Electroencephalogr Clin Neurophysiol. (1990) 76:148–64. 10.1016/0013-4694(90)90213-4 1697243

[86] Rohrbaugh JW Varner JL Paige SR Eckardt MJ Ellingson RJ. Event-related perturbations in an electrophysiological measure of auditory sensitivity: effects of probability, intensity and repeated sessions. Int J Psychophysiol. (1990) 10:17–32. 10.1016/0167-8760(90)90041-B 2269644

[87] Makeig S Galambos R. The CERP: event related perturbation in steady-state responses. In: Basar E Bullock T, editors. Brain Dynamics: Progress and Perspectives. Berlin: Springer. (1989). p. 375–400. 10.1007/978-3-642-74557-7_30

[88] Rockstroh B Müller M Heinz A Wagner M Berg P Elbert T. Modulation of auditory responses during oddball tasks. Biol Psychol. (1996) 43:41–55. 10.1016/0301-0511(95)05175-9 8739613

[89] Ross B Pantev C. Auditory steady-state responses reveal amplitude modulation gap detection thresholds. J Acoust Soc Am. (2004) 115(5 Pt 1):2193–206. 10.1121/1.1694996 15139631

[90] Ross B Herdman AT Pantev C. Stimulus induced desynchronization of human auditory 40-Hz steady-state responses. J Neurophysiol. (2005) 94:4082–93. 10.1152/jn.00469.2005 16107530

[91] Motomura E Inui K Kawano Y Nishihara M Okada M. Effects of sound-pressure change on the 40 Hz auditory steady-state response and change-related cerebral response. Brain Sci. (2019) 9:203. 10.3390/brainsci9080203 31426410 PMC6721352

[92] Sugiyama S Kinukawa T Takeuchi N Nishihara M Shioiri T Inui K. Change-related acceleration effects on auditory steady-state response. Front Syst Neurosci. (2019) 13:53. 10.3389/fnsys.2019.00053 31680884 PMC6803388

[93] Bartha-Doering L Deuster D Giordano V am Zehnhoff-Dinnesen A Dobel C. A systematic review of the mismatch negativity as an index for auditory sensory memory: from basic research to clinical and developmental perspectives. Psychophysiology. (2015) 52:1115–30. 10.1111/psyp.12459 26096130

[94] Näätänen R Gaillard AW Mäntysalo S. Early selective-attention effect on evoked potential reinterpreted. Acta Psychol (Amst). (1978) 42:313–29. 10.1016/0001-6918(78)90006-9 685709

[95] Näätänen R Jacobsen T Winkler I. Memory-based or afferent processes in mismatch negativity (MMN): a review of the evidence. Psychophysiology. (2005) 42:25–32. 10.1111/j.1469-8986.2005.00256.x 15720578

[96] Shelley AM Ward PB Catts SV Michie PT Andrews S McConaghy N. Mismatch negativity: an index of a preattentive processing deficit in schizophrenia. Biol Psychiatry. (1991) 30:1059–62. 10.1016/0006-3223(91)90126-7 1756198

[97] Catts SV Shelley AM Ward PB Liebert B McConaghy N Andrews S. Brain potential evidence for an auditory sensory memory deficit in schizophrenia. Am J Psychiatry. (1995) 152:213–9. 10.1176/ajp.152.2.213 7840354

[98] Chen TC Hsieh MH Lin YT Chan PS Cheng CH. Mismatch negativity to different deviant changes in autism spectrum disorders: a meta-analysis. Clin Neurophysiol. (2020) 131:766–77. 10.1016/j.clinph.2019.10.031 31952914

[99] Pekkonen E Jousmäki V Könönen M Reinikainen K Partanen J. Auditory sensory memory impairment in Alzheimer's disease: an event-related potential study. NeuroReport. (1994) 5:2537–40. 10.1097/00001756-199412000-00033 7696598

[100] Skosnik PD Krishnan GP O'Donnell BF. The effect of selective attention on the gamma-band auditory steady-state response. Neurosci Lett. (2007) 420:223–8. 10.1016/j.neulet.2007.04.072 17556098

[101] Bidet-Caulet A Fischer C Besle J Aguera PE Giard MH Bertrand O. Effects of selective attention on the electrophysiological representation of concurrent sounds in the human auditory cortex. J Neurosci. (2007) 27:9252–61. 10.1523/JNEUROSCI.1402-07.2007 17728439 PMC6673135

[102] Sugiyama S Kinukawa T Takeuchi N Nishihara M Shioiri T Inui K. Tactile cross-modal acceleration effects on auditory steady-state response. Front Integr Neurosci. (2019) 13:72. 10.3389/fnint.2019.00072 31920574 PMC6927992

[103] Tan HR Gross J Uhlhaas PJ. MEG-measured auditory steady-state oscillations show high test–retest reliability: a sensor and source-space analysis. NeuroImage. (2015) 122:417–26. 10.1016/j.neuroimage.2015.07.055 26216274

[104] Giard MH Peronnet F. Auditory-visual integration during multimodal object recognition in humans: a behavioral and electrophysiological study. J Cogn Neurosci. (1999) 11:473–90. 10.1162/089892999563544 10511637

[105] Senkowski D Molholm S Gomez-Ramirez M Foxe JJ. Oscillatory beta activity predicts response speed during a multisensory audiovisual reaction time task: a high-density electrical mapping study. Cereb Cortex. (2006) 16:1556–65. 10.1093/cercor/bhj091 16357336

[106] Tseng HH Bossong MG Modinos G Chen KM McGuire P Allen P. A systematic review of multisensory cognitive-affective integration in schizophrenia. Neurosci Biobehav Rev. (2015) 55:444–52. 10.1016/j.neubiorev.2015.04.019 25956248

[107] Baum SH Stevenson RA Wallace MT. Behavioral, perceptual, and neural alterations in sensory and multisensory function in autism spectrum disorder. Prog Neurobiol. (2015) 134:140–60. 10.1016/j.pneurobio.2015.09.007 26455789 PMC4730891

